# The Third Extracellular Loop of Mammalian Odorant Receptors Is Involved in Ligand Binding

**DOI:** 10.3390/ijms232012501

**Published:** 2022-10-18

**Authors:** Tammy Shim, Jody Pacalon, Won-Cheol Kim, Xiaojing Cong, Jérémie Topin, Jérôme Golebiowski, Cheil Moon

**Affiliations:** 1 Department of Brain Sciences, Daegu Gyeongbuk Institute of Science and Technology, Daegu 711-873, Korea; 2 Institut de Chimie de Nice UMR7272, Université Côte d’Azur, CNRS, 06108 Nice, France; 3 Institut de Génomique Fonctionnelle, Université de Montpellier, CNRS, INSERM, CEDEX 05, 34094 Montpellier, France

**Keywords:** odorant receptors, ECL3, ligand selectivity, molecular modeling, functional assays

## Abstract

Mammals recognize chemicals in the air via G protein-coupled odorant receptors (ORs). In addition to their orthosteric binding site, other segments of these receptors modulate ligand recognition. Focusing on human hOR1A1, which is considered prototypical of class II ORs, we used a combination of molecular modeling, site-directed mutagenesis, and in vitro functional assays. We showed that the third extracellular loop of ORs (ECL3) contributes to ligand recognition and receptor activation. Indeed, site-directed mutations in ECL3 showed differential effects on the potency and efficacy of both carvones, citronellol, and 2-nonanone.

## 1. Introduction

Mammals rely on their sense of smell to assess the volatile chemical environment. Smell information is decoded by chemical interactions between odorants and G protein-coupled odorant receptors (ORs) expressed in olfactory neurons present in the nasal cavity. While these transmembrane proteins are the cornerstone of chemical recognition by our neurons, perireceptor events can modify the chemical composition of odorants before reaching the ORs [1]. For example, the enzyme carboxyl esterase has been shown to influence odor recognition by altering the chemical function [2]. Odorant-binding proteins also modulate the chemical signal by helping to solubilize odorants or playing the role of scavengers [3].

Nonetheless, the coding of a perceived odor by the olfactory system relies on a combinatorial code where ORs are differentially activated by odorants and where one odorant can activate multiple ORs [4,5]. The subtle interactions between ORs and odorants at the molecular level remain extremely difficult to rationalize. In fact, very subtle modifications in the chemical structure of a molecule can drastically alter its odor [6,7]. Similarly, mutations within an OR gene might strongly alter smell perception [8].

ORs represent more than 3% of the whole proteome and belong to the class A G protein-coupled receptor (GPCR) family of proteins, which are responsible for transmitting signals through the cell membrane. The large number of ORs (~400 in humans and ~1100 in mice, for example) coupled with this combinatorial activation endows mammals with incredible discriminatory power [9,10]. GPCRs are one of the largest and most diverse membrane protein families. They adopt a typical architecture consisting of seven transmembrane helices (TM1 to TM7) linked by intracellular and extracellular loops (ICLs and ECLs, respectively). ECLs, while peripheral within the GPCR tertiary structure, are involved in numerous receptor functions such as ligand or protein recognition and receptor activation [11,12,13,14]. In most class A GPCRs, the internal binding site for the ligand is found at ca. 10 Å with respect to the extracellular side of the receptor. In the prototypical beta2-adrenergic receptor, multiple interactions have been described between the ligand and ECL3 during the process of ligand migration from the bulk solvent to the internal binding site [15]. In particular, ECL3 was suggested as a functional region important for ligand specificity. In a sub-class of mammalian ORs, namely, the class I ORs, we showed that the extracellular part of the receptor, notably ECL3, played the role of a vestibular binding site [16]. The sequence variability in the ECL3 is high between the two OR classes, which prevents us from making any conclusions on the role of ECL3 in class II ORs based on results from class I. This function of ECL3 in the class II OR has not been investigated until now.

In this article, we report on the role of ECL3 in class II ORs and show that ECL3 modulates ligand binding independent of the sequence variability between the two classes. We consider hOR1A1 as the prototypical class II OR. We thus conclude that ECL3 is involved in odorant selectivity in all mammalian ORs.

## 2. Results

### 2.1. The Highly Variable ECL3 Sequence Acts as a Vestibular Binding Pocket for Ligands

**Figure 1:**
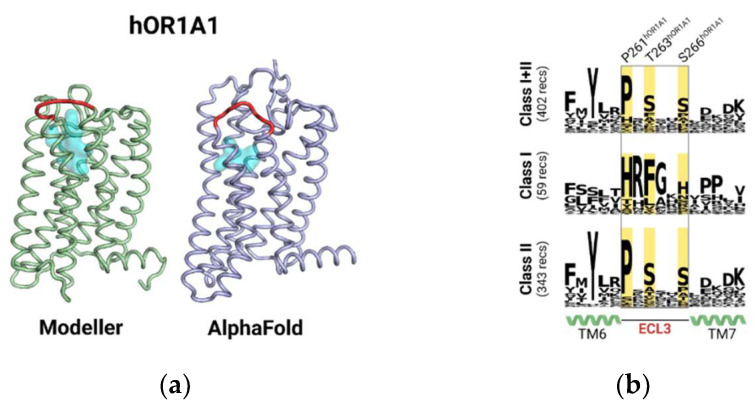
(**a**) Structure of hOR1A1 from homology modeling (Modeller) compared to that obtained from AlphaFold2 (AlphaFold). In both structures, the third extracellular loop (ECL3) (shown in red) was predicted to be close to the orthosteric binding cavity, shown as a cyan surface. (**b**) Conservation analysis of ECL3 sequences of both classes of human odorant receptors and the highlight of hOR1A1 specific ECL3 sequence.

In class A GPCRs, ECL3 connects the extracellular parts of TM6 and TM7. Up to now, no structure of hOR1A1 has been experimentally solved. A 3D modeled structure was built using a previously established protocol [17,18]. An alternative model was obtained using AlphaFold [19]. Figure 1a depicts the two modeled 3D structures of hOR1A1. The overall structure of the two models is conserved with an RMSD of 3.5 Å on the backbone. In general, the main difference occurs in the unstructured segment of the receptor. In both models, ECL3 was located ca. 4 Å toward the extracellular side of the orthosteric binding cavity. In hOR1A1, ECL3 comprised five/six residues (P261/L262 to S266) and showed an unfolded 3D structure. The vicinity of ECL3 and the binding cavity was consistent with a potential role of ECL3 as a vestibular binding site as already observed by us on class I ORs, and by others on the beta2-adrenergic receptor and muscarinic M2 receptors [15,16,20].

From a sequence point of view, the conservation analysis and its decomposition into class I and class II ORs revealed that the positions in ECL3 were conserved in class II ORs and formed a P261xSxxS motif (Figure 1b). In human ORs, the proline (here P261) was conserved at 72.3%, S263 at 46.5%, and S266 at 44.1%. In hOR1A1, the sequence reads PLTNYS. They were not only different from those found in class I but also different from any other class A GPCRs.

**Figure 2:**
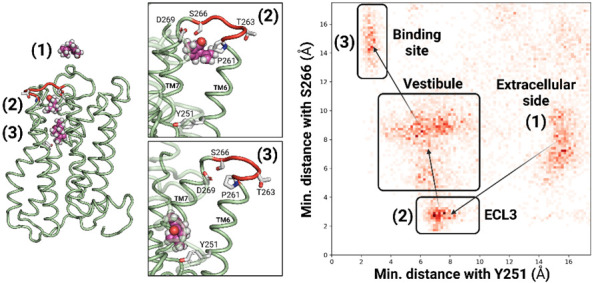
Entry of (−)-carvone inside receptor hOR1A1. The ligand is initially located outside the receptor (1). It then migrates to the cradle of the orthosteric binding cavity (2,3), as indicated by Y251^6.48^. During this process, the ligand interacts with several residues from ECL3 (indicated in red). Contour map of (−)-carvone migration as the minimum distance from S266 (taken as the distance from ECL3) and minimum distance from Y251^6.48^ (taken as the distance from the cradle of the cavity). All replicas were considered. The three highlighted basins show the ligand’s largest density.

The involvement of ECL3 in the binding process was assessed by a molecular dynamics (MD) simulation where (−)-carvone, a strong ligand for hOR1A1 [21,22], was initially located within the bulk solvent. When all replicas were aggregated, the binding mechanism could be decomposed into a three-step process. Figure 2 depicts the main events identified during the trajectories and highlights the density of (−)-carvone in the protein with respect to both residue Y251^6.48^ (bottom of the orthosteric cavity) and S266^ECL3^. Starting from the bulk solvent, the ligand rapidly approached the extracellular segment of the receptor and initiated contact with ECL3 before reaching the orthosteric binding site. The density map confirmed the three regions in which the ligand spent more time. Upon the binding process, the ligand interacted with various residues in ECL3, highlighting this loop’s involvement in the ligand’s entry within the receptor.

In our homology model, P261^ECL3^ is the first residue of ECL3 and acts as a (−)-carvone contact point with the receptor after ca. 100 nanoseconds. The ligand then interacted with the hydrophobic residues Leu262^ECL3^ and diffused towards the orthosteric binding site, engaging H-bonds with Ser266^ECL3^ (Figure 2, position 2). Finally, the ligand interacted with Asp269^7.34^ before entering the binding cavity (Figure 2, on the left, position 3), i.e., at less than 2.5 Å of Y251^6.48^ (6.48 refers to the Ballesteros–Weinstein notation) [23,24]. To assess the role of amino acids from ECL3 in the receptor recognition process, in vitro experiments were also performed.

### 2.2. In Vitro Functional Assays Highlight the Differential Interactions with Diverse Ligands

**Figure 3:**
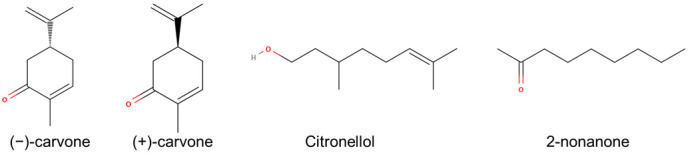
Chemical structure of four agonists of hOR1A1.

Based on the results of both the conservation analysis and the MD simulations, several amino acids from ECL3 seem to be involved in the binding process. To investigate the specific chemical functions of amino acids in ECL3 upon ligand binding, we designed mutant ORs with various (small, charged, lipophilic, or aromatic) properties, and assessed their response to four different ligands (Figure 3).

**Figure 4:**
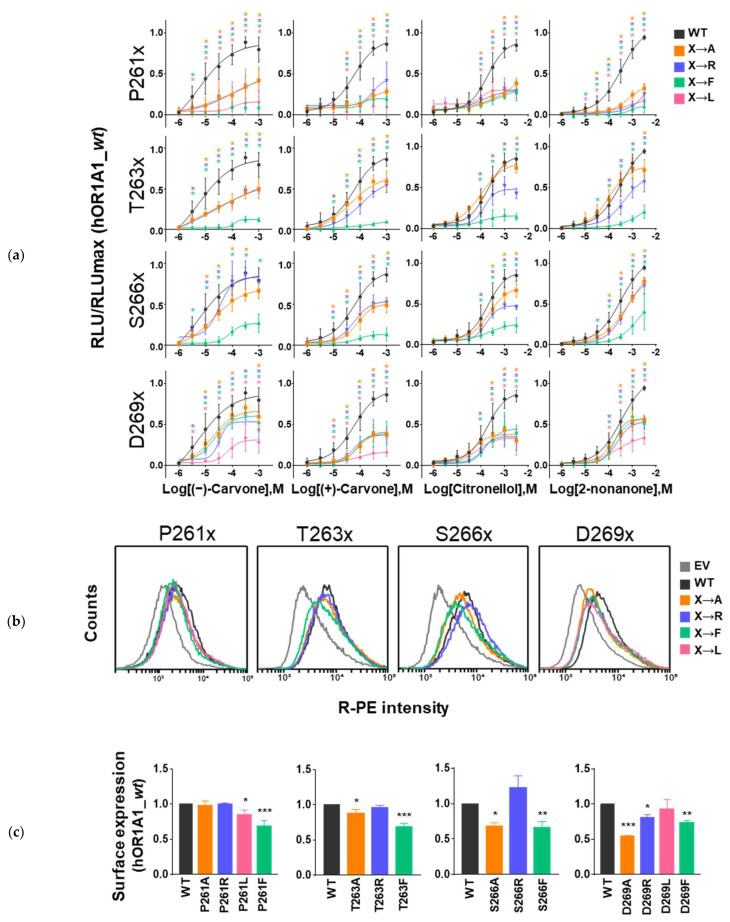
In vitro data of hOR1A1 and mutant ORs. (**a**) In vitro dose–response curves of four ligands (−)-carvone, (+)-carvone, citronellol, and 2-nonanone towards *wt* hOR1A1 and mutant ORs at positions P261, T263, S266, and D269. (*) indicates the response value is significantly different compared to *wt* hOR1A1 (one-way ANOVA, followed by a Dunnett test; * *p* < 0.05). (RLU = relative luminescence unit) (**b**) The fluorescence intensity of the R-phycoerythrin (R-PE) signal of *wt* hOR1A1 and mutant ORs at positions P261, T263, S266, and D269. (**c**) Normalized graph of cell-surface expression of ECL3 mutant ORs against *wt* hOR1A1 (one-way ANOVA, followed by a Dunnett test; * *p* < 0.05, ** *p* < 0.01, and *** *p* < 0.001).

All the mutant ORs investigated in this study were confirmed to be expressed at the membrane surface and showed some basal activities, confirming that they remain functional (Figure 4b,c and Supplementary Materials). However, it appeared that the expression level of the mutant ORs was differentially affected compared to the *wt* OR (Figure 4b,c). The presence of a phenylalanine residue systematically resulted in a decrease in surface expression for all four mutant ORs. Mutation to an alanine residue at all three hydrophilic positions (T263^ECL3^, S266^ECL3^ and D269^7.34^) also decreased surface expression of the receptor, whereas this mutation had no effect on expression for the P261A mutant OR. Surprisingly, the presence of a positive charge at positions P261^ECL3^, T263^ECL3^, and S266^ECL3^ did not affect surface expression. The same modification at position D269^7.34^ induced a decrease in surface expression. Overall, we observed that bulky amino acids in ECL3 resulted an increased basal activity (Figure S3) independent of surface expression.

The *wt* OR exhibited a dose-dependent increase in cAMP levels in response to four ligands ((−)-carvone, (+)-carvone, citronellol, and 2-nonanone) known as bona fide agonists in vitro (Figure 3 and Figure 4a) [21,23,25]. Mutations at positions P261, T263, S266, and D269 differentially affected the receptor response to agonists (Figure 4a).

MD simulations revealed interactions between the (−)-carvone and P261^ECL3^ early in the binding process. P261X mutant ORs (X = A, R, L, or F) did not show a dose-dependent response for all four ligands: their efficacy and potency were significantly reduced (Figure 4a). Mutations affected the agonist-induced response differently: P261A and P261R remained sensitive to the most potent ligand, (−)-carvone, whereas the receptors’ responses were negligible for the other three ligands. Mutations to a more hydrophobic and bulkier residue, such as leucine or phenylalanine (Figure 4a pink and green, respectively), eliminated the response to all four ligands. These data show that mutations at position P261 differentially affect the response to ligands and reinforce the hypothesis of the initial interaction between P261 and ligands during the binding process.

T263 and S266 are two small and polar conserved residues in the ECL3 PxSxxS motif, which strongly interacted with (−)-carvone during the MD simulations. Notably, mutations at position 263 have been reported to affect OR2AG1 response to agonist [23]. Interestingly, mutations at these positions had a different impact on the receptor response to the 4 agonists. The mutations into apolar (A), charged (R), or aromatic (F) residues were—as for P261—also associated with differential modification of the receptor response to ligands in vitro.

The T263F mutation abolished the receptor response to all four ligands (Figure 4a, second row, green curves). The presence of positively charged residues systematically decreased the efficacy of all four ligands (Figure 4a, second row, blue curve). Finally, all four ligands were differentially affected by introducing an alanine at position 263. The response to 2-nonanone and citronellol remained unchanged compared to *wt* OR, while the efficacy of the two enantiomers of carvone decreased. This observation suggested a specific interaction of residue T263 with both carvone enantiomers.

Mutations at position S266 also induced changes in efficacy. Surprisingly, (−)-carvone efficacy for the S266R mutant OR remained unchanged (Figure 4a, 3rd row, black curve vs. blue curve), whereas it was systematically reduced for the other ligands. The S266A and S266F mutant ORs decreased the efficacy of all four ligands. As for the other three residues investigated, introducing a phenylalanine residue almost abrogated the receptor response.

D269^7.34^ was not predicted to belong to ECL3 in both the Modeller and AlphaFold models. It was located at the upper extremity of TM7. One can observe in Figure 4c that surface expression of the mutant ORs was systematically decreased except for D269L, in line with the role of the extracellular part of TM7 in the activation mechanism [26]. Compared to the mutant ORs at ECL3, the impact of the mutation on receptor response to agonist stimulation was more systematic. The mutations to A, R, or F induced a similar decrease in the receptor response for the four studied ligands. The D269L mutant OR showed a much more pronounced decrease in response to agonist stimulation except for citronellol (Figure 4a, last line, pink color). This systematic trend suggests that D269 seems to be involved in the dynamics of receptor activation rather than in ligand-binding recognition.

All these experiments underline the importance of ECL3 residues during binding. It can be observed that the presence of a phenylalanine at the position studied systematically altered the expression level. Furthermore, the response of the phenylalanine mutant ORs to the four ligands was abrogated, except for D269F, which remained sensitive to agonist stimulation.

In general, the mutations did not alter the EC50. When a dose–response curve was observed, the EC50 never shifted more than one order of magnitude in concentration. The mutations clearly affect the efficacy of the ligands. Finally, the major takeaway of our study is that from a general point of view, the efficacy and potency of ligands are differentially affected by the mutations in ECL3.

## 3. Discussion

Odorant receptors, as all class A GPCRs, are structured with a seven-transmembrane domain where helices are connected by three extra- and three intra-cellular loops. Among these loops, the short ECL3 connects TM6 to TM7 and shows a large sequence variability. Several works have already evaluated the role of this very poorly conserved ECL3. In particular, they assessed its essential contribution to the ligand-binding process of the beta2-adrenergic receptor or the muscarinic M2 receptor [15,20]. The active role of ECL3 in ligand binding is consistent with the close vicinity of ECL3 and the orthosteric binding site. This has been, for example, illustrated in some peptide-binding class A GPCRs, where the peptide directly interacts with both ECL3 and the orthosteric binding pocket [27,28]. Note that ECL3 structures in various peptide-bound class A GPCR are much more variable than ECL1 or ECL2, suggesting that ECL3 plays a modulation role rather than a ligand recognition role [29]. All in all, the role of ECL3 in class A GPCRs is not only structural but it seems to modulate the ligand’s recognition in combination with the binding site.

In class I ORs, a sub-family of mammalian ORs, we previously proposed that ECL3 functions as a vestibule site and contributes to ligand binding [16]. In this work, we hypothesized a similar involvement of ECL3, although the conserved residues are significantly different between the two sub-families of ORs. Through a joint approach combining molecular modeling, heterologous functional expression, site-directed mutagenesis, and functional assays on hOR1A1—a prototypical class II OR—we have evaluated the role of ECL3 in ligand binding. In class II ORs, ECL3 is based on the conserved P^261^ xS^263^ xxS^266^ motif. Various mutant ORs at positions 261, 263, and 266 showed differential responses to four agonists belonging to various chemical families compared to *wt*.

Meanwhile, additional mutant ORs at position D269^7.34^ (at the junction between ECL3 and TM7) showed a conserved modulation regardless of the type of substituted amino acid. Molecular dynamics simulations of a homology model of hOR1A1 in interaction with (−)-carvone sampled structures where the ligand was in regular contact with ECL3 prior to entering the orthosteric binding cavity.

hOR1A1 has been extensively studied for its property of discriminating the two enantiomers of carvone [23]. In a former study combining homology modeling, site-directed mutagenesis, and functional expression studies, the authors identified eleven positions on the transmembrane segments involved in the chiral recognition of carvone. However, our results reveal that the two enantiomers of carvone are similarly affected by mutations in ECL3.The conserved residues in ECL3, although involved in agonist recognition, are apparently not involved in chiral discrimination in hOR1A1.

Finally, our results suggest that ECL3 in hOR1A1 plays a similar role in ligand binding as observed in the beta-2-adrenergic receptor, the M2 muscarinic receptor, and class I ORs. This study therefore reconciles all previous studies and demonstrates a similar function of ECL3 on ligand binding in all class A GPCRs, independent of the ECL3 sequence. The large variability in the ECL3 sequence among class A GPCR subfamilies is consistent with the diverse chemical space associated with these receptors, which can bind highly polar or highly lipophilic ligands. It could become an interesting and alternative target for allosteric ligand design.

## 4. Materials and Methods

### 4.1. In Silico Experiments

#### 4.1.1. Molecular Modeling

hOR1A1 models were created using Modeller 9.25 [30]. Our previous protocol was described by De March et al. with four experimental structures as templates (PDB codes: 1U19, 3ODU, 2YDV, and 2LNL) [18]. Cysteine residues 74 with 156, and 146 with 166 were assigned to form two disulfide bonds [31]. The best-generated models were selected based on the DOPE score, and one model was finally chosen based on visual inspection. The hOR1A1 AlphaFold model was retrieved from the AlphaFold structure database [32,33].

Modeller and AlphaFold models have a 3.5 Å RMSD (calculated on the backbone of TMs). Both models nicely superimpose concerning TM6 and TM7. The distance between Y251^6.48^ and ECL3’s amino acids is comparable in the hOR1A1 Modeller and AlphaFold model (15.7/17.4 Å, 19.0/20.2 Å, 19.0/19.4 Å and 14.7/14.3 Å for residues P261, T263, S266 and D269, respectively, for Modeller/AlphaFold). While ECL3 is made up of almost identical residues, its structure is predicted with low confidence by AlphaFold (pLDDT between 50 and 70). The model built by homology was kept for the molecular dynamics simulations.

#### 4.1.2. Molecular Dynamics Preparation

As the N-terminal of GPCR is not resolved in the template we used, the N-terminal part of the hOR1A1 Modeller model was discarded until residue E24. Propka3 was used to predict protonation states of the protein at a target pH 6.5 [34]. The extremities of the model were capped accordingly. hOR1A1 orientation in its membrane was determined using the OPM server [35]. Three (−)-carvone molecules were added in different orientations on the extracellular side at 5–10 Å of the top of the receptor. The system was embedded into a POPC-only model membrane using PACKMOL-Memgen [36]. The simulation box was completed using TIP3P water molecules and neutralized using K^+^ and Cl^−^ ions with a final concentration of 0.15 M. The total system comprised 49,536 atoms in a 7 × 10^5^ Å³ periodic box.

#### 4.1.3. Molecular Dynamics Protocol

Molecular dynamics simulations were performed with the sander and pmemd.cuda modules of AMBER18 [37], with the ff14SB force field for the proteins and the lipid14 forcefield for the membrane. (−)-carvone parameters were generated by calculating partial atomic charges with the HF/6–31G* basis set using Gaussian 09 [38]. The obtained electrostatic potential was fitted by the RESP program [39]. The other parameters were taken from the general Amber force field 2 (gaff2). Bonds involving hydrogen atoms were constrained using the SHAKE algorithm and long-range electrostatic interactions were handled using particle mesh Ewald. The cut-off for non-bonded interaction was set to 10 Å. Each system was first minimized with the AMBER sander module, with 5000 steps of steepest descent and then 5000 steps of the conjugate gradient with a 50 kcal∙mol^−1^∙Å² harmonic potential restraint on the protein. A second minimization of the same length without restraint was applied. The systems were then thermalized from 100 to 310 K for 10,000 steps (restraining the protein and ligands with a 200 kcal∙mol^−1^∙Å² harmonic potential). Each system underwent 50,000 steps of equilibration in the NPT ensemble and 1 bar (restraining the protein and ligands with a 15 kcal∙mol^−1^∙Å² harmonic potential) before the production phase. During the equilibration and production phase, the temperature was kept constant in the system at 310 K using a Langevin thermostat with a collision frequency of 5 ps^−1^. To increase sampling, all 3 (−)-carvone molecules were constrained in a sphere of 30–40 Å radius, centered on the center of mass of the Thr89 hOR1A1 (with a potential of 10 kcal∙mol^−1^∙Å²).

The system stability was evaluated from the root mean square deviation (RMSD) evolution computed on the receptor backbone. For the 8 replicas, the receptors underwent small fluctuations (RMSD < 4 Å), showing that they remained correctly folded during microsecond simulations (Figure S1). Binding events occurred in Rep1 (stable) and Rep3 (partial entry) (Figure S2). Cpptraj and Pytraj v2.02.dev0 were used for distance and RMSD analysis [40].

### 4.2. In Vitro Experiments

#### 4.2.1. Cell Culture

HEK293T cell line was obtained from ATCC (#CRL-3216, ATCC, Manassas, VA, USA) and cultured in Dulbecco’s modified Eagle’s medium (DMEM; #10-017-CV, Corning, NY, USA) plus 10% fetal bovine serum (#16000-044, Thermo Fisher Scientific, Waltham, MA, USA) and penicillin/streptomycin (#15140122, Thermo Fisher Scientific, Waltham, MA, USA) at 37 °C with 5% CO_2_.

#### 4.2.2. DNA Constructs, Site-Directed Mutagenesis, and Gene Transfection

The full genomic DNA sequence of *hOR1A1* was obtained from HEK293T cells using cloning primers (5′-GCA CGC GTA TGA GGG AAA ATA ACC AGT C-3′ and 5′-GCG CGG CCG CTT ACG AGG AGA TTC TCT T-3′). PCR product was subcloned to LUCY-FLAG-Rho tagged pCI mammalian expression vector using MluI and NotI restriction enzyme. The pCI vector, which is the Rho sequence-tagged at N-terminal, RTP1s-pCI, G_olf_-pCI, and Ric8b-pCI was a kind gift from Dr. Matsunami (Duke U., Durham, NC, USA). A cleavable leucine-rich 17 amino acid signal peptide (LUCY; MRPQILLLLALLTLGLA) and FLAG sequences were tagged in front of the Rho sequence to promote or detect the functional expression on the plasma membrane, respectively [41]. pHIV-EGFP was a gift from Bryan Welm and Zena Werb (Addgene plasmid #21373). pGloSensor^TM^-22F cAMP Plasmid was purchased (#E2301, Promega, Madison, WI, USA). Site-directed mutant ORs were generated through the QuikChange PCR protocol [42] using the mutagenic oligonucleotide primers, designed individually according to the desired mutation. The sequence of plasmids was confirmed through BigDye Terminator v3.1 Cycle Sequencing Kit (Thermo Fisher Scientific, Waltham, MA, USA). Furthermore, JetPRIME (#114-75, PolyPlus-transfection, Illkirch-Graffenstaden, France) was used for gene transfection. For FACS experiments, the OR or mutant OR, RTP1s, Ric8b, G_olf_, and pHIV-EGFP plasmid were transfected, and for cAMP luminescence assay, the pGloSensor^TM^-22F cAMP plasmid was transfected instead of the pHIV-EGFP plasmid.

#### 4.2.3. cAMP Luminescence Assay

The cAMP luminescence assay was performed using the GloSensor^TM^ cAMP assay (Promega, Madison, WI, USA) by following the manufacturer’s protocol. In brief, the media of the transfected HEK293T cells on the 96-well white plate were changed to CO_2_ independent media (#18045088, Thermo Fisher Scientific, Waltham, MA, USA) with GloSensor^TM^ cAMP Reagent (#E1291, Promega, Madison, WI, USA) before exposure to solvent or odorants. Each odorant was diluted into CO_2_ independent media and used to treat each well. The endpoint of the luminescence level was measured with a SpectraMax L Microplate Reader (Molecular Devices, San Jose, CA, USA). Data were analyzed through Microsoft Excel and GraphPad Prism. To compare OR responses plate to plate, empty pCI vector and wild-type OR were always included as a control. To read the basal activity of each OR, the values of at least six wells were averaged in the absence of odorants. The experiments were repeated twice to measure the odorant-induced OR activity, and each condition was triplicated. The measured luminescence value was further corrected by subtracting the value for the lowest response to each odorant of that receptor. The basal activity and odorant-induced responses were normalized to that of wild-type OR.

#### 4.2.4. Functional Expression

The functional expression of OR on the plasma membrane was measured through fluorescent-activated cell sorting (FACS) as previously described [43]. To summarize, transfected HEK293T cells on the six-well plates were gently detached from the plate using Cellstripper (#25-056-CI, Thermo Fisher Scientific, Waltham, MA, USA). The cells were incubated with Rho4D2 antibody (#ab98887, Abcam, Cambridge, MA, USA) for an hour with gentle rotation. After the cells were washed twice with washing and staining solution (2% fetal bovine serum (FBS) and 15M NaN_3_ in 500 mL PBS), they were incubated for 30 min with gentle rotation to attach R-PE-conjugated secondary antibody (#715-116-151, Jackson ImmunoResearch Inc., West Grove, PA, USA). Lastly, the cells were washed twice, and the dead cells were labeled with 7-AAD (#SML1633, Merck, Darmstadt, Germany). The fluorescence of immunolabeled cells was detected and analyzed using BD Accuri^TM^ C6 Plus Flow Cytometer (BD Biosciences, San Jose, CA, USA). After removing 7-AAD signal-positive dead cells, the intensity of the R-PE signal among the GFP-positive cells was measured and plotted.

## Data Availability

Not applicable.

## Supplementary Materials

The following supporting information can be downloaded at: https://www.mdpi.com/article/10.3390/ijms232012501/s1.
