# Supplementary material for: The Third Extracellular Loop of Mammalian Odorant Receptors Is Involved in Ligand Binding
Source: Int J Mol Sci. 2022 Oct 18;23(20):12501. doi: 10.3390/ijms232012501 (PMC9604345; doi:10.3390/ijms232012501)
Supplement: Supplementary file 1 [file ijms-23-12501-s001.zip › ijms-1884216-supplementary.pdf]

International Journal of
Molecular Sciences

MDPI

The Third Extracellular Loop of Mammalian Odorant Receptors Is Involved in Ligand Binding

Tammy Shim [1,†], Jody Pacalon [2,†], Won-Cheol Kim [1], Xiaojing Cong [3], Jérémie Topin [2,*], Jérôme Golebiowski [1] and Cheil Moon [1,*]

Supplementary materials

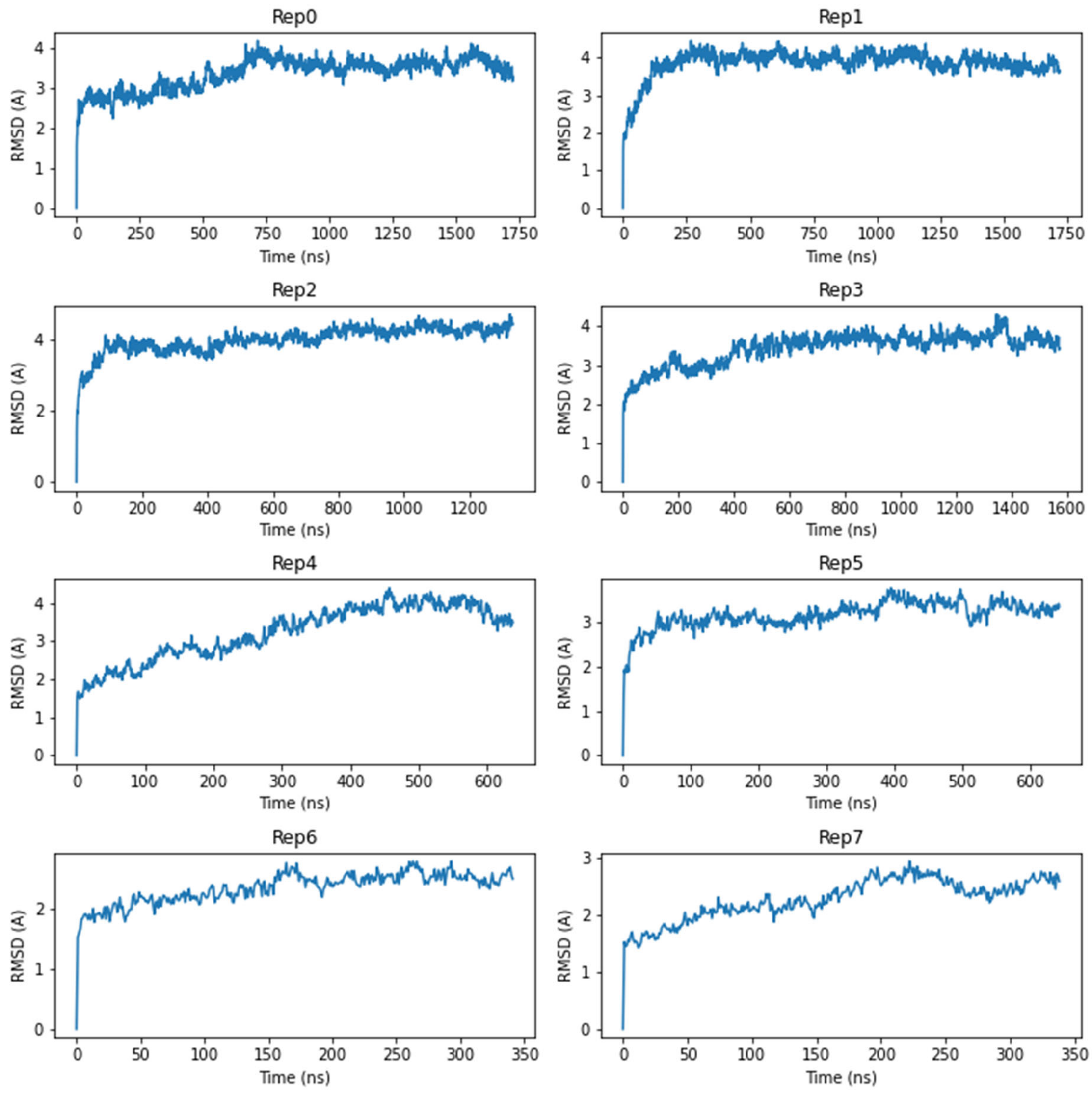

Supplementary Figure S1. Time evolution of the Root Mean Square Deviation (RMSD) of the 8 replicas of hOR1A1 studied. The RMSD is computed on N, Cα, C backbone atoms with respect to the structure of the initial model.

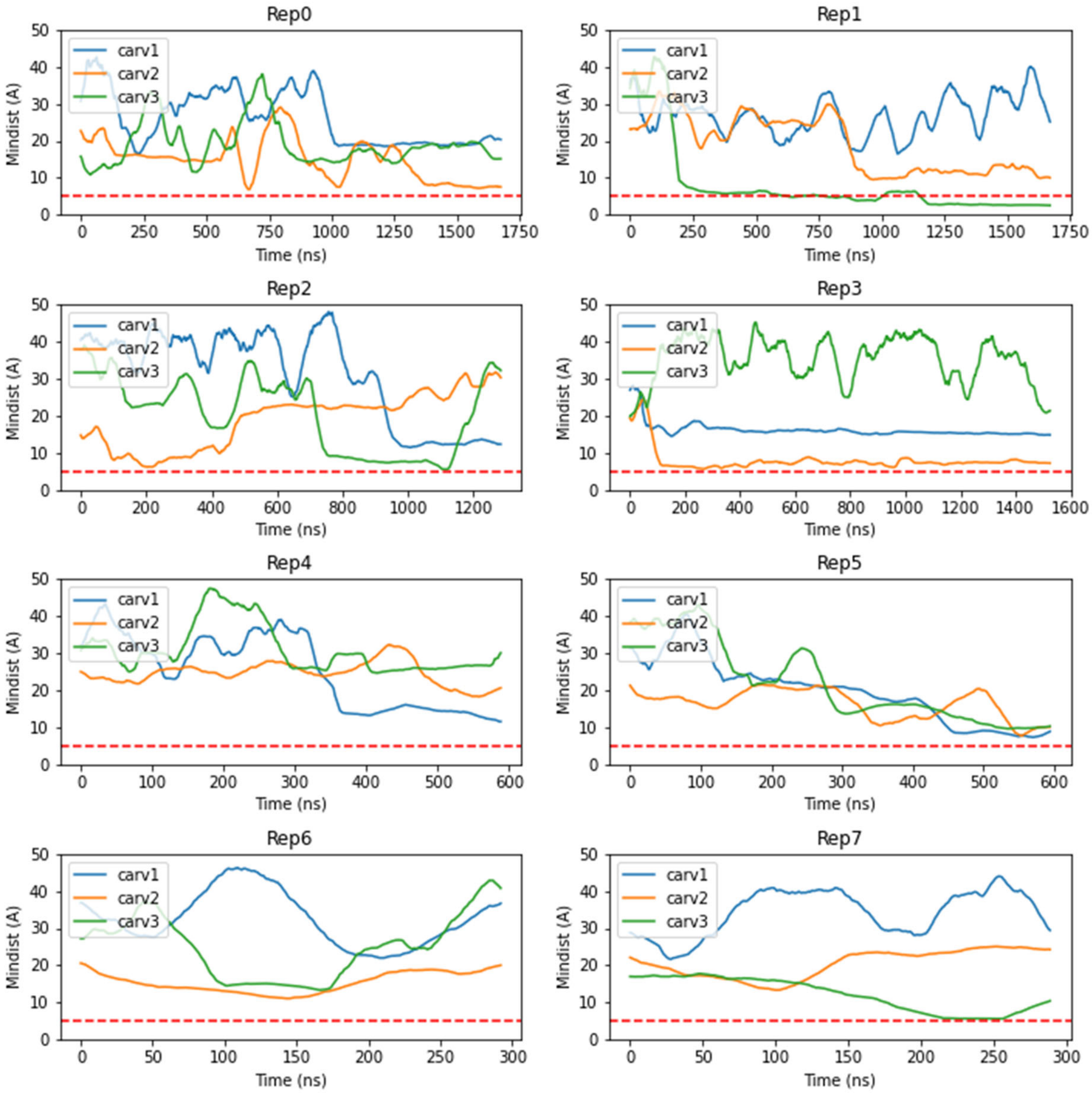

Supplementary Figure S2. Evolution of the distance between the center of mass of the three individual (−)-carvone molecules (in blue, yellow and green) and the center of mass of residue Y251 (cradle of the binding cavity). The dotted red line at 5 Å indicates the threshold at which we consider an interaction between a molecule and residue Y251.

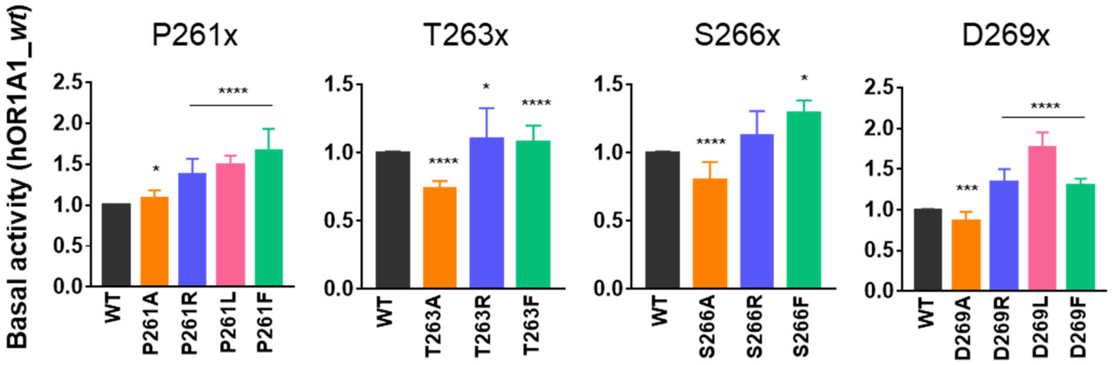

Supplementary Figure S3. Basal activity graph of *wt* hOR1A1 and mutants at positions P261, T263, S266, and D269. (Two-tailed

unpaired t-test; *$p<0.05$, ***$p<0.001$, and ****$p<0.0001$)
